# Refining Surgical Corridors with Whole Brain Tractography: A Case Series

**DOI:** 10.7759/cureus.1672

**Published:** 2017-09-10

**Authors:** Hosniya Zarabi, Anil Roy, Avilasha Jha, Gustavo Pradilla

**Affiliations:** 1 Neurosurgery, Emory University School of Medicine

**Keywords:** whole brain tractography, diffusion tensor imaging, automatic seeding

## Abstract

Recent advancements in automated diffusion tensor imaging (DTI) and whole brain tractography (WBT) may be of great use to the neurosurgeon in selecting surgical corridors that can minimize disruption of surrounding white matter tracts. This is especially important in cases where the lesion displaces white matter tracts and traditional operative approaches may inadvertently violate these fibers. Here, we present automated DTI seeding and WBT as a practical and efficient means for preoperative surgical planning, in an effort to spare white matter tracts that may be displaced by a variety of lesions and may be vulnerable during surgery. We retrospectively reviewed the records of seven patients with various intracranial lesions, who underwent preoperative magnetic resonance imaging (MRI) with automated DTI analysis. These images were used to guide operative planning so that we could select white matter corridors that would allow for minimal damage to vulnerable fiber tracts. The patients had various pathologies, ranging from neoplasms to intracranial hemorrhage, in a number of different intracranial locations. All the patients underwent preoperative intracranial imaging with post-processing of these images to generate white matter tracts. These images were then used to design an appropriate surgical approach that would minimize injury to white matter tracts. For the patients with neoplasms, all were totally or near-totally resected with a stability of symptoms postoperatively. In the case of the patient with intracranial hemorrhage, the hematoma was evacuated, with significant improvement in the postoperative period. Automated DTI seeding and WBT, which have become increasingly prevalent in recent years, can be of significant use to the neurosurgeon for preoperative planning. Their application is especially important in cases where white matter tracts are displaced by the lesion in question and are put at risk of injury during surgery. Using WBT to design customized surgical approaches appropriate to the case at hand can be of immense value in preserving these white matter tracts, minimizing postoperative deficits, and improving surgical outcomes. Further studies are needed to validate these results and better define their applicability to other regions and pathologies.

## Introduction

Diffusion tensor imaging (DTI) is an advanced magnetic resonance imaging (MRI) technique that allows for non-invasive evaluation of white matter tracts. It relies on the diffusion of water as a function of spatial location and is based on principles of fractional anisotropy (FA), as well as eigenvector directions in three-dimensional space [1]. The technique, which has become increasingly prevalent in recent years, is quite advantageous for neurosurgeons, allowing for evaluation of white matter tracts surrounding a particular lesion as well as preoperative planning. Traditionally, a method called “manual seeding” was used to generate the information contained in tractographic images. This method entailed the expertise of a neuroradiologist to identify a region of interest and analyze fractional anisotropy data in order to generate white matter tracts that were then overlapped onto standard T1 and T2 sequences. This method requires significant processing time and is associated with inter-user variability and biased tract visualization [2-3]. 

In recent years, automated seeding and whole brain tractography (WBT) has been advanced to streamline the application of DTI for pre-operative neurosurgical planning. Automated seeding relies on post-processing software that allows for data extraction from all voxels to generate tracts in two or three dimensions. While there is certainly variability among the software packages available from different vendors and centers, these automated analyses appear to generally outperform manual processing in the efficiency of tract generation [4].

White matter tracts in the cerebrum may be divided into three categories: projection fibers, which run vertically and spread information from the cerebrum to the body; commissural fibers, which extend horizontally and transmit information between the two hemispheres; and association fibers, which connect different structures within the same hemisphere. The internal capsule and optic radiations comprise the major projection fibers, while the corpus callosum, as well as the anterior and posterior commissures, are the main commissural fibers in the brain. Association fibers include longer lobar connections as well as shorter gyral connections. The major association fibers include the superior longitudinal fasciculus (SLF), inferior longitudinal fasciculus (ILF), arcuate fasciculus (AF), uncinate fasciculus (UC), inferior fronto-occipital fasciculus (IFO), and the cingulate fasciculus (CF). It is important to note that injury to these association fibers may not be readily evident on a routine neurological exam, but may have long-term neurological sequelae and significant associated morbidity. Ideomotor apraxia and spatial neglect have been associated with SLF injury [5]. Disruption of the UF can cause language deficits, affect, and behavioral changes [6]. Deficits associated with ILF injury include visual agnosia and prosopagnosia [7]. Minimizing injury to white matter tracts is thus crucial to preserving function and minimizing morbidity from neurosurgical procedures. In an effort to achieve this overarching goal, automated DTI seeding and WBT may be used for preoperative planning to identify surgical corridors that would minimize the transgression of these white matter fibers when pursuing a lesion of interest.

The displacement of white matter fibers can vary significantly based on the underlying pathology. In our experience, most anterior basal ganglia hemorrhages have a predictable displacement of white matter fibers, and routine tractography may not be necessary. These anterior basal ganglia hemorrhages displace the internal capsule medially and the SLF laterally, making them very favorable for an atraumatic anterior surgical corridor evacuation. On the other hand, neoplasms tend to displace white matter fibers less predictably. Thus, the approach for tumors requires a more thorough review of the DTI images, so that adequate customization of the surgical corridor may be achieved.

Here, we present our experience with the use of automated DTI seeding and WBT to identify surgical corridors that would be the least disruptive to perilesional white matter tracts and would consequently maximize preservation of function, while minimizing postoperative morbidity. Safe surgical corridors for a variety of lesions including neoplasms and an intracranial hemorrhage are presented.

## Case presentation

Methods

We retrospectively reviewed the records of seven patients with various intracranial lesions who underwent preoperative MRI imaging with automated DTI analysis. These images were used to guide operative planning so that we could select white matter corridors that would allow for minimal damage to vulnerable fiber tracts. The study was approved by an institutional review board (IRB).

Image acquisition

All patients underwent a clinical MRI of the brain on a 1.5 Tesla scanner, which included DTI as well as post-contrast, thin cut volumetric T1- weighted images. The exception was the patient in case four, whose images were obtained at a different hospital using a 3.0 Tesla scanner, and who did not have any post-contrast MRI images. T1-weighted images were obtained using the following parameters: TR = 1900 ms, TE = 3.52 ms, T1 = 900 ms, flip angle 9°, field-of-view (FOV) = 256 x 256 mm, slide thickness = 1 mm. For all patients, DTI was performed using b values of 0, and 1000 s/mm2. The following parameters were used to obtain thirty gradient directions: TR = 8600 ms, TE = 89 ms, FOV = 240 x 240 mm, slice thickness = 2.0 mm.

Synaptive automated processing

The aforementioned DTI images were processed using the Synaptive Brightmatter platform, an automated processing system equipped with planning capabilities to generate WBT (Synaptive Medical, Toronto, Canada, 2015). Using a proprietary algorithm that makes use of a deterministic 4th order Runge-Kutta interpolator, Synaptive's algorithm generates all fiber tracts, which are then overlaid over T1 or T2 MRI sequences to aid in surgical planning. This process is entirely automated and does not require the guidance of a radiologist for manual seeding.

Approach selection

In order to minimize damage to white matter tracts, the surgical approach traversing the fewest number of white matter fibers was selected. The selected approaches were determined by manually demarcating the lesion on each slice so that a three-dimensional volumetric image of the entire lesion and any surrounding tracts could be generated. The surgical corridor was then constructed while taking into account any patient positioning and cosmetic constraints.

Results

We identified seven cases that made use of preoperative tractography for surgical decision making. Postoperative tractography data were available for most but not all cases.

Case 1

**Figure 1:**
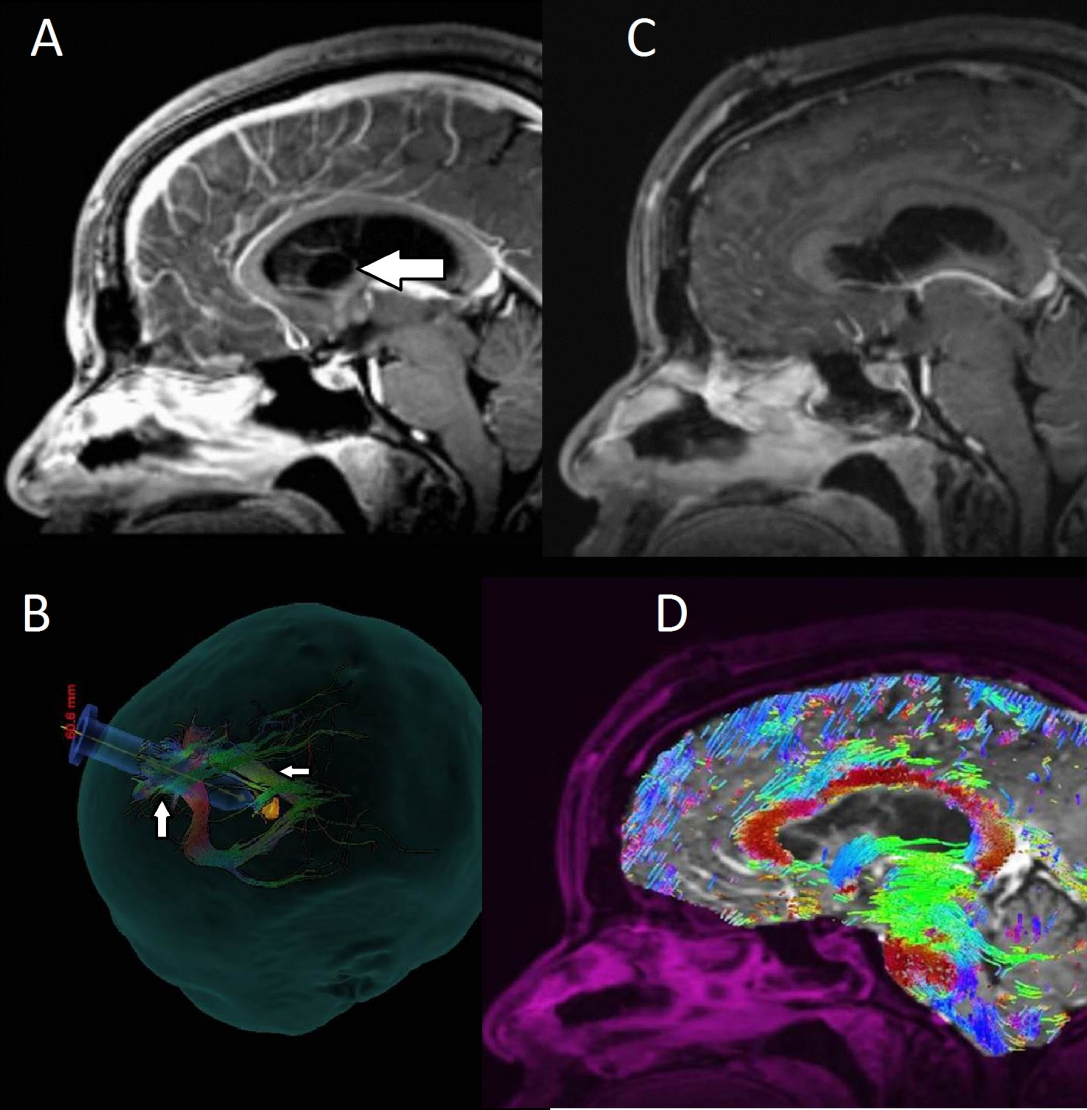
Preoperative and postoperative images for patient one with a colloid cyst A: Preoperative images reveal a colloid cyst (arrow) in the anterior part of the third ventricle and bilateral foramina of Monro with associated hydrocephalus and thinning of the corpus callosum. B: WBT allowed us to determine an almost parafascicular route along the SLF (upward arrow) that avoided the internal capsule (leftward arrow) fibers. C: Postoperative images showing resection of the colloid cyst. D: Preservation of the surrounding projection and association white matter tracts after resection of the colloid cyst.

The patient is a 30-year-old female who presented with a headache that persisted for two weeks after a ground-level fall. A computed tomography (CT) scan of her head revealed a hyperdense lesion at the foramen of Monro with associated hydrocephalus. The MRI showed the lesion to be a subcentimeter non-enhancing lesion, likely representing a colloid cyst, in the anterior part of the third ventricle and bilateral foramina of Monro. There was associated hydrocephalus, as well as elevation and thinning of the corpus callosum (Figure 1 A). Post-processing of these MRI images (Figure 1 B) was performed as noted above, and a transsulcal approach was selected. WBT allowed us to tailor the approach to an almost parafascicular route along the SLF, avoiding the internal capsule. A right frontal craniotomy for microsurgical resection of the colloid cyst with a port-based approach using the pre-selected corridor was performed uneventfully. The patient remained neurologically intact postoperatively, and her cyst was successfully removed (Figure 1C) while preserving the surrounding projection and association white matter tracts (Figure 1 D). The diagnosis of a colloid cyst was confirmed on final pathology.

Case 2

**Figure 2:**
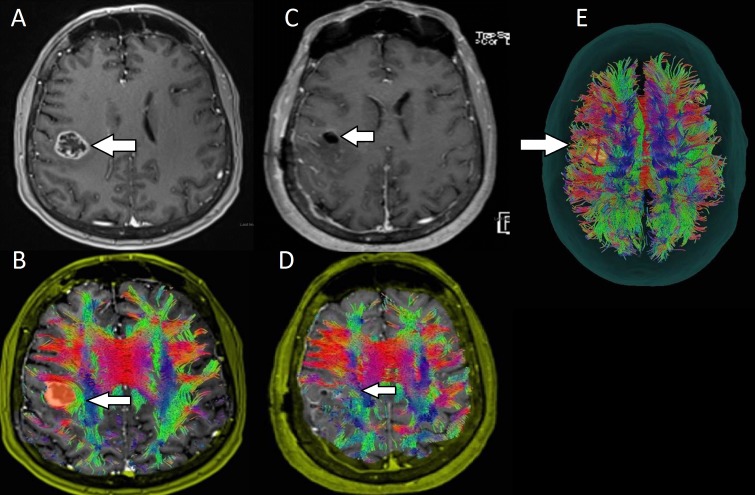
Preoperative and postoperative images for patient two with a left-sided glioblastoma multiforme (glioblastoma multiform) A: Preoperative images showing a contrast-enhancing lesion (arrow) centered at the gray-white junction in the right periorlandic region with surrounding edema. B: DTI showing medial displacement of the SLF (arrow). C: Postoperative images showing gross total resection of the mass (arrow), which was determined to be GBM. D: Preservation of the SLF (arrow) after resection of the tumor. E: A transsulcal exposure (arrow) was deemed to be the least disruptive surgical corridor in approaching this lesion.

The patient is a 62-year-old female whose chief complaint of left facial droop and slurred speech prompted a CT scan of the head. This scan showed a mass centered in the gray-white junction in the right peri-rolandic region with surrounding edema. Further work up did not show any evidence of systemic malignancy. MRI of the brain with DTI showed a 19 x 18 mm contrast-enhancing lesion with surrounding vasogenic edema and 3 mm of right-to-left midline shift (Figure 2A). Post-processing of these images (Figure 2B) allowed us to visualize a transsulcal exposure as the least disruptive surgical corridor for approaching this tumor (Figure 2E). Medial displacement of the SLF was noted (Figure 2B). A right frontal craniotomy was performed and the tumor was resected uneventfully (Figure 2C) with preservation of white matter tracts (Figure 2D). The patient had transient worsening of her left grip strength postoperatively, which improved at the time of her six-week postoperative visit. Her final pathology was consistent with glioblastoma multiforme.

Case 3

**Figure 3:**
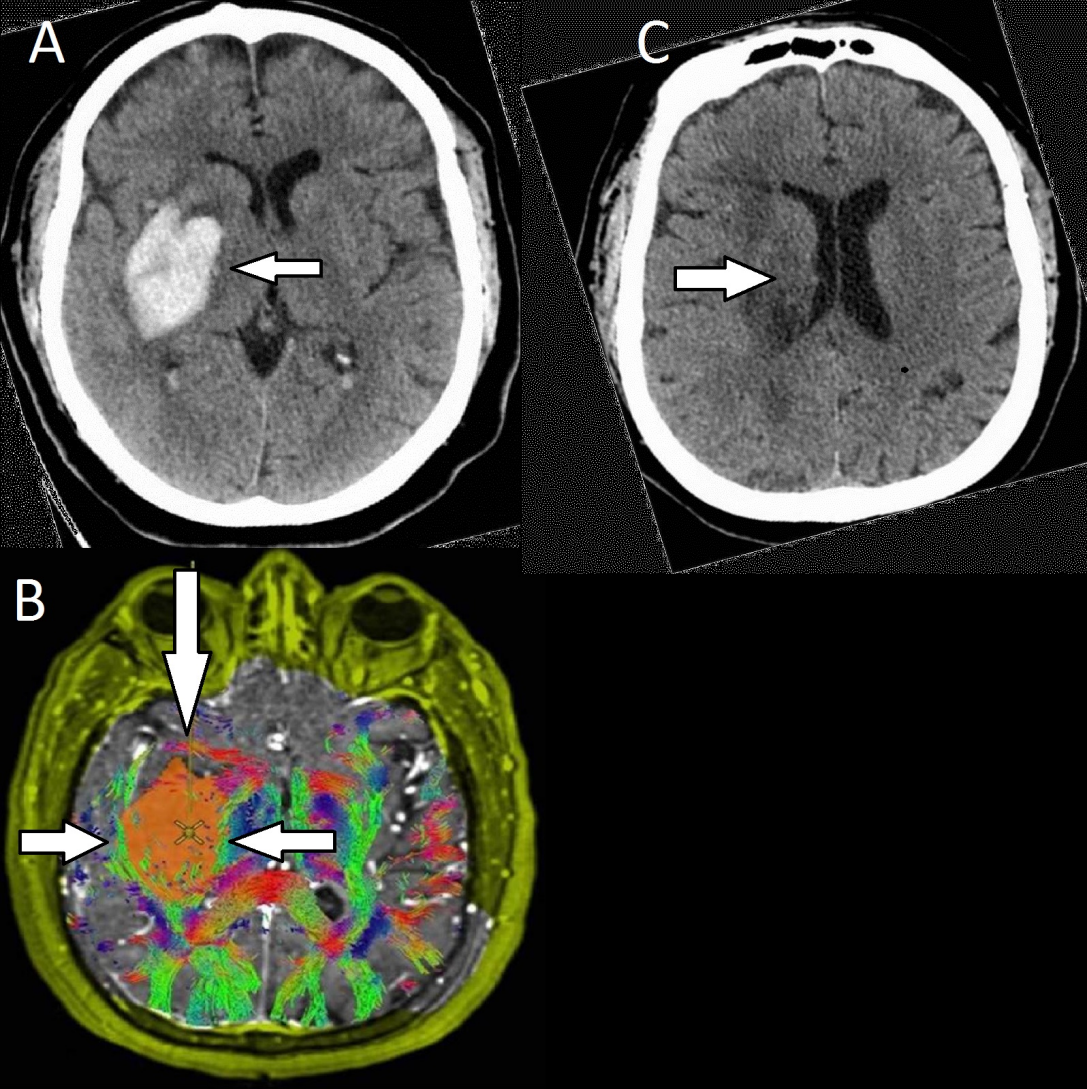
Preoperative and postoperative images for patient three with a basal ganglia hemorrhage A: CT scan of the head showed a 4.8 x 3.0 x 4.0 cm right intraparenchymal hemorrhage (arrow) with surrounding edema. B: DTI revealed medial displacement of the internal capsule (leftward arrow) and lateral displacement of the SLF (rightward arrow). An anterior corridor was used to approach the hematoma (downward arrow). C: A postoperative CT scan of the head showing evacuation of the hematoma (arrow).

The patient is a 73-year-old female with a history of uncontrolled type II diabetes and hypertension, who presented with left hemiparesis and was found to have a right basal ganglia hemorrhage. She was intubated due to declining mental status, and a CT scan showed a 4.8 x 3.0 x 4.0 cm right intraparenchymal hemorrhage with surrounding edema, partial effacement of the right lateral ventricle, right uncal medialization, and 5 mm of right-to-left midline shift (Figure 3A). No vascular etiology was noted on the CT angiogram. The patient then underwent an MRI with tractography, which revealed medial displacement of the internal capsule and lateral displacement of the SLF (Figure 3B). The patient underwent a supraorbital forehead crease approach to engage the long axis of the clot in the corridor, avoiding the internal capsule, and remaining parafascicular to the SLF. The subcortical space was cannulated with a port-based approach, and the hematoma was evacuated (Figure 3C). Postoperative WBT images are not available for this patient, but postoperative CT scan showed a gross total clot evacuation. The patient made a significant recovery with her mental status, and at her last clinic follow-up visit, she was noted to be initiating some spontaneous movements on her left side. The patient was also conversant, although with some slurred speech.

Case 4

**Figure 4:**
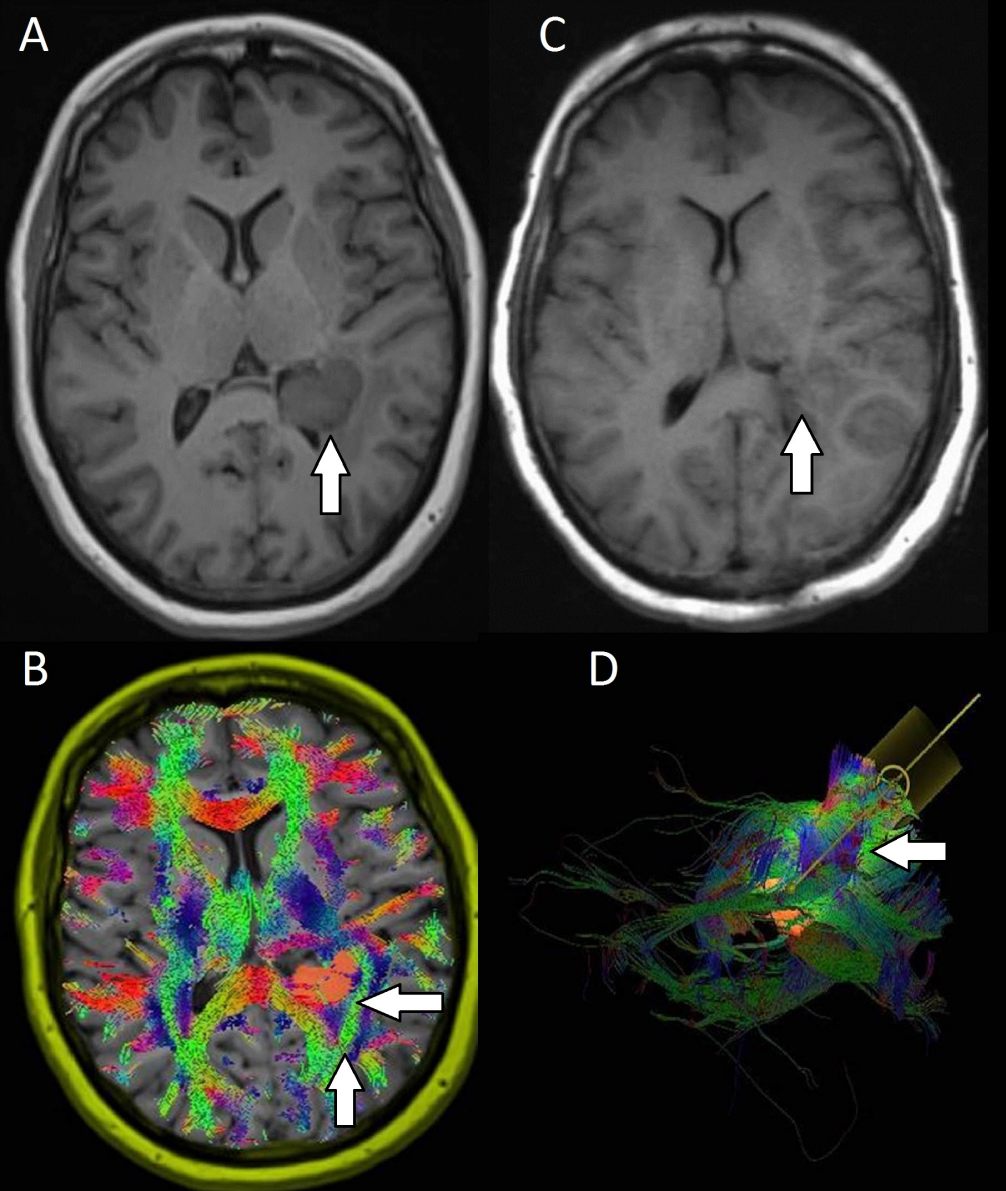
Preoperative and postoperative images for patient four with a left atrial meningioma A: Preoperative MRI showed a diffusion-restricting mass (arrow) in the atrium of the left ventricle, causing mass effect in the left temporoparietal region. B: DTI showed that the lesion was displacing the left ILF (partially visualized, leftward arrow), front-occipital (not visualized here) and optic radiation (partially visualized, upward arrow) fibers. C: Postoperative images showing gross total resection of the mass, which was determined to be a WHO Grade I meningioma. D: A variant of the superior parietal lobule approach was designed to engage the association fibers in a parafascicular fashion (arrow) so as to minimize injury.

The patient is a 54-year-old female who presented with gait imbalance and mild cognitive decline. Intracranial imaging showed a diffusion restricting mass in the atrium of the left ventricle, thus causing mass effect in the left temporoparietal region (Figure 4A). Post-processing of these images showed that the lesion was displacing the left ILF and the inferior frontooccipital fibers, as well as the optic radiations (Figure 4B). The surgical corridor selected was a variant of the superior parietal lobule approach designed specifically to engage the association fibers in a parafascicular fashion and minimize injury (Figure 4D). This patient underwent a port-based resection, and gross total resection of her tumor was achieved (Figure 4C). The patient did have some worsening contralateral visual and motor deficits postoperatively, but these improved significantly at the time of her last clinic visit. Postoperative edema prevented the generation of reliable postoperative tractography. The final pathology was consistent with a World Health Organization (WHO) grade I meningioma.

Case 5

**Figure 5:**
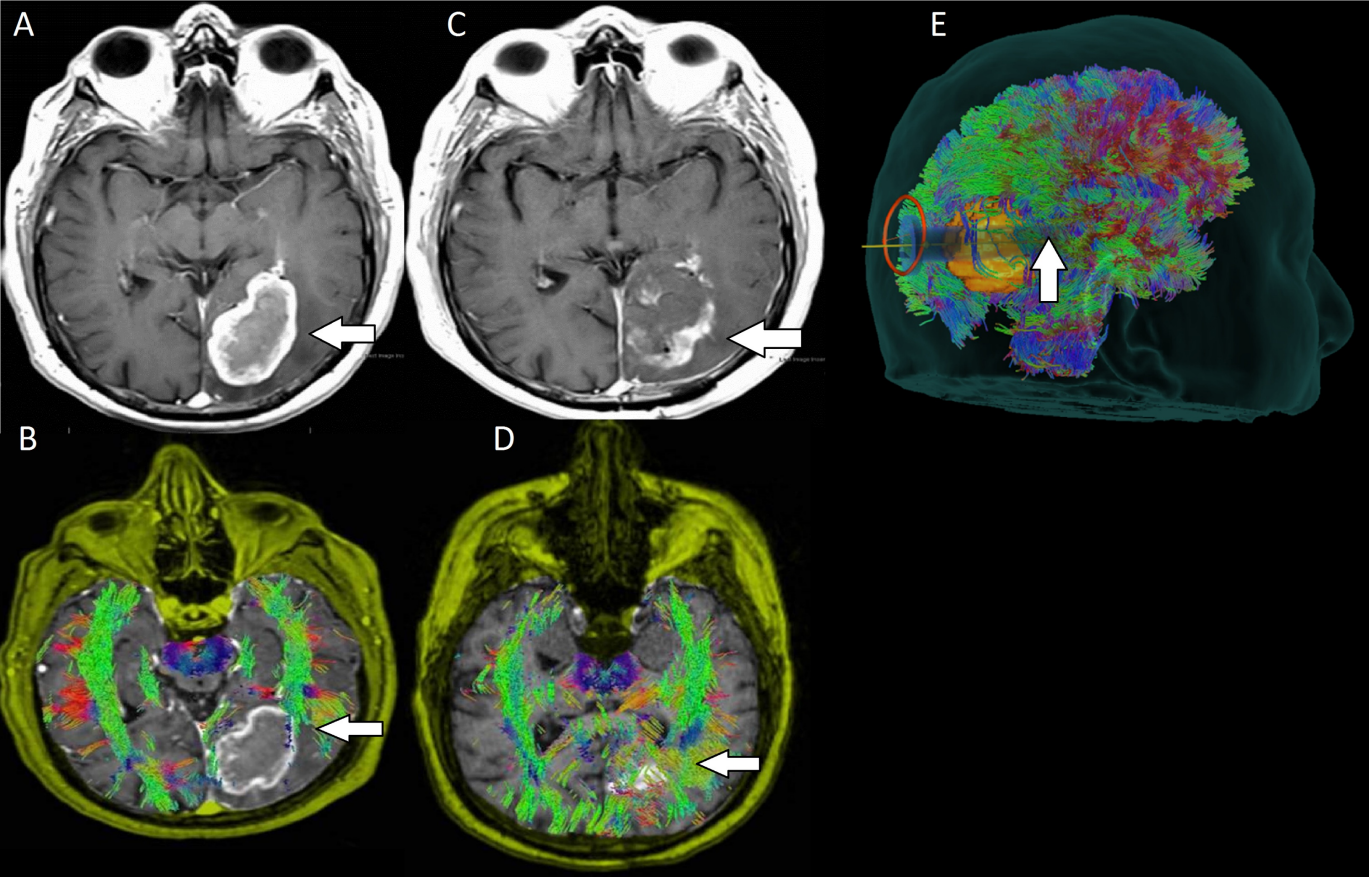
Preoperative and postoperative images for patient five with an occipital glioblastoma multiforme (GBM) A: Preoperative images revealed a heterogeneously ring-enhancing left occipital lesion (arrow). B: DTI showed lateral and inferior displacement of the optic radiations (arrow). Note that the presence of edema prevents the complete reconstruction of these fibers. C: Postoperative images showing near total resection of the mass, which was determined to be a GBM (arrow). D: Postoperatively, the optic radiations were noted to have a more anatomical location (arrow). E: A medial surgical corridor was constructed to minimize injury to the surrounding optic radiations (arrow).

The patient is a 59-year-old male who presented with a two-week history of headache, nausea, and difficulty with vision in his right visual field. MRI revealed a heterogeneously ring-enhancing left occipital lesion (Figure 5A). Pre-operative WBT revealed lateral and inferior displacement of the optic radiations (Figure 5B) and a medial surgical corridor was constructed. (Figure 5E). The patient underwent a port-based occipital craniotomy, with postoperative imaging revealing a near-total resection (Figure 5C). Postoperative tractography revealed the optic radiations to be in a more anatomical position (Figure 5D). The patient’s neurological exam remained stable after surgery. Final pathology was consistent with glioblastoma multiforme (GBM).

Case 6

**Figure 6:**
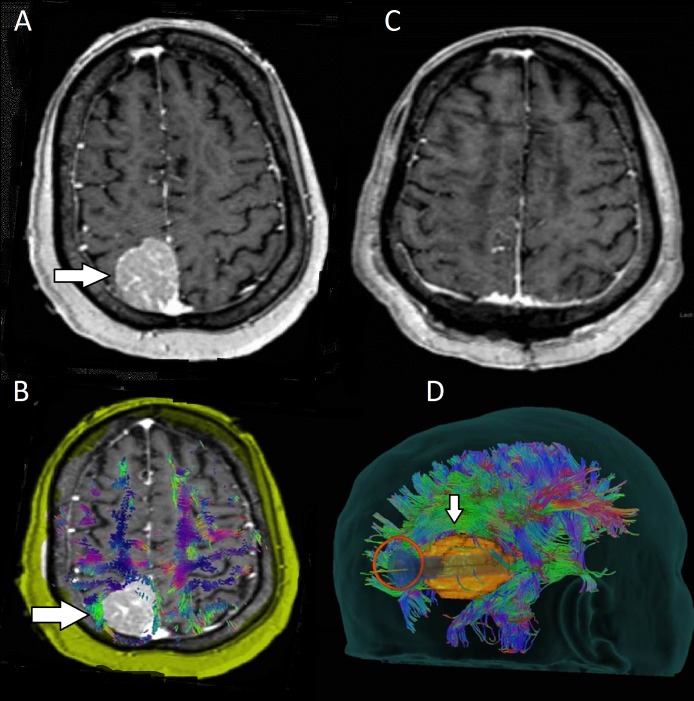
Preoperative and postoperative images for patient six with a posterior parietal meningioma A: Preoperative images revealed an extra-axial mass (arrow) in the right posterior parietal lobe with significant local mass effect. B: Lateral displacement of the SLF was noted on WBT (arrow). C: Postoperative images showed gross total resection of the mass, which was determined to be a WHO Grade I meningioma. D: A posterior surgical corridor was designed to avoid injury to the SLF (arrow).

The patient is a 56-year-old female with a past medical history of stroke without any residual deficits as well as multiple sclerosis, who presented with altered mental status, left-sided twitching, and several months of left leg weakness. The MRI of the brain showed a 3.5 x 2.5 cm extra-axial mass in the right posterior parietal region with significant local mass effect (Figure 6A). Elevated fluid-attenuated inversion recovery (FLAIR) signal changes were seen in the periventricular region and in the subcortical white matter consistent with her history of white matter disease. WBT revealed lateral displacement of the SLF (Figure 6B). The patient underwent a parietal craniotomy, and gross total resection of the mass was achieved (Figure 6C). She had a significant improvement in her leg weakness postoperatively. Her final pathology was consistent with a WHO Grade I meningioma.

Case 7

**Figure 7:**
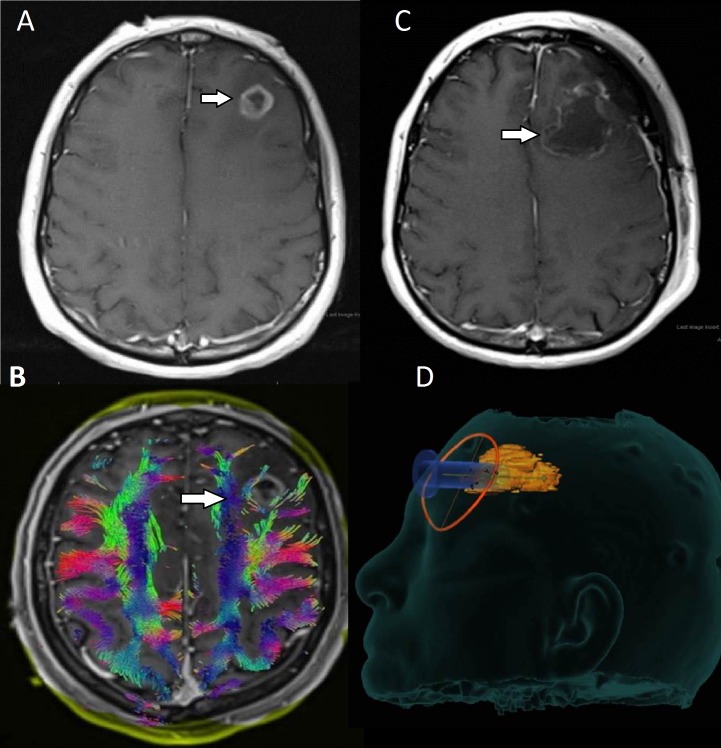
Preoperative and postoperative images for patient seven with a left frontal GBM A: Preoperative image showed a peripherally enhancing mass (arrow) in the left frontal lobe with surrounding edema as well as an area of enhancement along the frontal horn of the right lateral ventricle (not seen here). B: WBT revealed displacement of the SLF (not visualized), IFO (not visualized), and the anterior thalamic radiations (rightward arrow). C: Postoperative images showed gross total resection of the mass, which was determined to be glioblastoma multiforme (arrow). D: An anterior surgical corridor was designed to avoid the surrounding white matter tracts.

The patient is a 58-year-old male who initially presented after a motor vehicle accident that had occurred because he suffered a seizure while driving. He sustained multiple orthopedic injuries from the accident. A CT scan of his head, followed by an MRI with automated DTI seeding, showed a 1.5 x 1.5 x 2.5 cm peripherally enhancing mass in the left frontal lobe with surrounding edema, as well as a 1 cm enhancement along the frontal horn of the right lateral ventricle (Figure 7A). The tumor appeared to displace the SLF, IFO, and the anterior thalamic radiations (Figure 7B). The patient underwent a left frontotemporal craniotomy, and a port-based system was used to cannulate to the depth of the tumor using an anterior corridor. The tumor was resected in total. The cortical incision was then extended anteriorly, and the remainder of the lesion, as well as the area of enhancement along the right lateral ventricle, was resected (Figure 7C). Postoperatively, the patient had full strength in his bilateral upper extremities. On presentation, his lower extremity strength was limited due to several orthopedic injuries that required external fixation. At his first follow-up, he had full strength in the left lower extremity, but his right lower extremity was still difficult to assess due to his orthopedic injuries, although he was able to move his toes well. The final pathology for his lesion was consistent with glioblastoma multiforme.

## Discussion

Image guidance is a critical component of modern neurosurgical practice, especially when dealing with deep-seated lesions. There has been tremendous recent interest in the use of DTI for additionally refining our surgical corridors so as to minimize damage to white matter tracts. Although traditional neuronavigation provides purely anatomical targeting, the use of DTI allows us to fine-tune approaches, avoiding critical white matter fibers [8].

Conventional DTI relies on manual seeding, which requires the expertise of a neuroradiologist to generate the images [9]. This method suffers from reproducibility and inter-user reliability [2, 3]. More recently, automatic seeding by various software platforms and WBT have allowed for a more efficient and systematic approach to generating information that the neurosurgeon may use to identify the most fiber-sparing surgical approach. While not all white matter tracts can be spared, with WBT the neurosurgeon may identify an approach that would be least disruptive to these tracts, thus presumably allowing for better surgical outcomes. In the cases presented above, automated seeding without the input of a neuroradiologist was used to identify surgical corridors that would best spare perilesional white matter tracts.

WBT is especially important in those cases where the lesion has disrupted established anatomical boundaries, as standard anatomical approaches may not take into account critical white matter tracts [10]. In the case of damage to such fibers, the resulting deficits may not be readily identified on a routine bedside neurological exam, but their long-term impact on patients can cause profound postoperative morbidity. Although the superior parietal lobule and transtemporal approach have been used with reports of good outcomes, routine neurological assessment cannot reliably detect deficits caused by damage to complex association fibers. For example, damage to the SLF, which has been associated with ideomotor apraxia and spatial neglect, would require detailed neuropsychological testing to establish its integrity [5]. The application of WBT is especially useful in the case of neoplasms, where the tumors tend to displace white matter tracts less predictably than lesions (such as intracranial hemorrhages). The neurosurgeon may use automated DTI seeding preoperatively to customize a surgical corridor that would minimize the transgression of perilesional white matter fibers, while still allowing for adequate resection of the lesion in question. While tumors require a more customized surgical approach, given their tendency to displace white matter tracts in an unpredictable fashion, a more standard approach can be used for intracranial hemorrhages as per our experience. We have routinely been employing an anterior parafascicular trajectory for anterior basal ganglia hemorrhages as they tend to displace white matter tracts in an expected pattern. Of the three basic approaches (anterior, posterior, and lateral), the anterior approach appears to be the mainstay for all basal ganglia hemorrhages as this approach engages the space between the SLF and the CF in a manner that is parallel, rather than perpendicular to these fibers. Thalamic hemorrhages require a posterior approach through the superior parietal sulcus. In these cases, visualization of the vertical rami of the SLF, optic radiations, and posterior arcuate fasciculus is critical, and DTI imaging is recommended. Lobar intracerebral hematomas that affect the dominant hemisphere with arcuate fasciculus or compromised ILF, as well as hemorrhages affecting the frontoparietal region with displacement or compromise of the corticospinal tract, can greatly benefit from pre-op DTI planning.

A key limitation of automated seeding analysis is that the presence of edema can hamper perilesional tract reconstruction. This can be a significant factor with regards to neoplasms that can have edema on preoperative and postoperative scans. There were patients in our series where postoperative tractography was not reliable due to an expected edema.

Here, we have presented seven cases where automated DTI and WBT were used to define safe surgical corridors in an effort to minimize iatrogenic injury to surrounding white matter tracts. While we presented a variety of lesions within various intracranial regions, further studies are required to validate these results as well as further define their applicability to other pathologies and locations.

## Conclusions

Automated WBT can significantly enhance the surgical armamentarium available to the neurosurgeon and the efficiency of the preoperative planning workflow. Further evidence is needed to define if manual or automated seeding is the most optimal technique for tractography. Further studies are also needed to define if the use of DTI improves patient outcomes.
